# Expert Failure: Re-evaluating Research Assessment

**DOI:** 10.1371/journal.pbio.1001677

**Published:** 2013-10-08

**Authors:** Jonathan A. Eisen, Catriona J. MacCallum, Cameron Neylon

**Affiliations:** 1 University of California Davis, Davis, California, United States of America; 2 Public Library of Science, Cambridge, United Kingdom

## Abstract

It is unlikely that there is any single objective measure of merit, so research assessment therefore requires new multivariate metrics that reflect the context of research, regardless of discipline.

Funding organisations, scientists, and the general public need robust and reliable ways to evaluate the output of scientific research. In this issue of *PLOS Biology*, Adam Eyre-Walker and Nina Stoletzki analyse the subjective assessment and citations of more than 6,000 published papers [1]. They show that expert assessors are biased by the impact factor (IF) of the journal in which the paper has been published and cannot consistently and independently judge the “merit” of a paper or predict its future impact, as measured by citations. They also show that citations themselves are not a reliable way to assess merit as they are inherently highly stochastic. In a final twist, the authors argue that the IF is probably the least-bad metric amongst the small set that they analyse, concluding that it is the best surrogate of the merit of individual papers currently available.

Although we disagree with some of Eyre-Walker and Stoletzki's interpretations, their study is important for two reasons: it is not only among the first to provide a quantitative assessment of the reliability of evaluating research (see also, e.g., [2]) but it also raises fundamental questions about how we currently evaluate science and how we should do so in the future.

Their analysis (see Box 1 for a summary) elegantly demonstrates that current research assessment practice is neither consistent nor reliable; it is both highly variable and definitely not independent of the journal. The subjective assessment of research by experts has always been considered a gold standard—an approach championed by researchers and funders alike [3]–[5], despite its problems [6]. Yet a key conclusion of the study is that the scores of two assessors of the same paper are only very weakly correlated (Box 1). As Eyre-Walker and Stoletzki rightly conclude, their analysis now raises serious questions about this process and, for example, the ∼£60 million investment by the UK Government into the UK Research Assessment Exercise (estimated for 2008), where the work of scientists and universities are largely judged by a panel of experts and funding allocated accordingly. Although we agree with this core conclusion and applaud the paper, we take issue with their assumption of “merit” and their subsequent argument that the IF (or any other journal metric) is the best surrogate we currently have.

**Box 1. The Error of Our Ways**

The analysis that Eyre-Walker and Stoletzki provides is clever and you should read it in full. The data on subjective assessment come from the Faculty1000 database [26], where published papers are rated by researchers, and from the scoring of previously published articles by a Wellcome Trust grant panel (the data are available in Dryad [11]). All the papers assessed were published in a single year (2005) and citation counts to the papers were collated from Google Scholar [27] in 2011. The five-year IFs from 2010 were used as they were over a similar timescale.

They reached their conclusions by partitioning the variation in the assessment scores and the number of citations that can be attributed either to “merit” or to “error” (i.e. the other possible factors that contribute to the variability). They also neatly sidestep defining merit independently, leaving it as whatever it is that makes someone score a paper highly.

It is already known that researchers and others rate papers more highly if they are from journals with higher IFs [2], but Eyre-Walker and Stoletzki carefully demonstrate the extent of this and control for the inflationary effect to reveal the crux of their study—that there is a woefully small correlation (*r*<0.2) between the different scores made by two assessors of the same paper (*N*>1,000). Moreover, in relation to “impact,” assessment scores explain even less of the variation in citations between papers (*r*≤0.15). As one of the reviewers of the article, Carl Bergstrom, stated:

“What it shows is not that evaluators fail to predict some objective measure of merit—it isn't clear, after all, what that objective measure of merit might even be. What this paper shows is that whatever merit might be, scientists can't be doing a good job of evaluating it when they rank the importance or quality of papers. From the (lack of) correlation among assessor scores, most of the variation in ranking has to be due to ‘error’ rather than actual quality differences.”

But the problems are potentially more insidious than this. Citations are also inflated by the IF (though there is much more variation in citations within than between journals; see [1] for their Figure 5). Once controlled for, however, the variation in citation counts *per se* that can't be explained by “merit” turns out to be even larger than the unexplained variance in the subjective scoring of scientists. The authors conclude that papers are therefore accumulating citations essentially by chance, a factor that helps to account for the low correlation between assessor score and citations. This also implies that we don't yet understand why some papers accumulate more citations than others, or what citation counts are telling us about individual articles in general.

Eyre-Walker and Stoletzki's conclusion that the IF is the best metric of the set they analyse is based purely on the fact that it is likely to have less bias or error associated with it than either subjective assessment by experts after publication or subsequent citations to individual papers. Their rationale is that IFs reflect a process whereby several individuals are involved in a decision to publish (i.e. reviewers), and simply averaging over a larger number of assessors means you end up with a stronger “signal” of merit. They also argue that because such assessment happens *before* publication, it is not influenced by the journal's IF. Even so, they accept that IFs will still be extremely error prone. If three reviewers contribute equally to a decision, and you assume that their ability to assess papers is no worse than those evaluating papers after publication, the variation between assessors is still much larger than any component of merit that might ultimately be manifested in the IF. This is not surprising, at least to editors, who continually have to juggle judgments based on disparate reviews.

First, and most importantly, their analysis relies on a clever setup that purposely avoids defining what merit is (Box 1). The lack of correlation between assessors is then interpreted as meaning that this hypothetical quantity is not being reliably measured. However, an alternative interpretation is that assessors are reliable at assessment, but are assessing *different things*. The lack of correlation, therefore, is a signal that “merit” is not a single measurable quantity. This is consistent with the finding that citation data are highly stochastic: the factors leading individuals to cite a paper (which the authors discuss) will also vary. Citations and subjective assessments of merit will therefore inevitably be the result of multivariate factors each with an associated variance that may act in different and nonlinear combinations—no wonder it looks like chance.

Second, the authors assume that the IF will be the best surrogate of merit because reviewers of papers *before* publication are less influenced by the journal (Box 1). They appreciate the many problems associated with the IF (e.g. [7]–[9]) and stress that it is not in any way a quantitative measure of merit. They acknowledge, for example, that an article in a journal with an IF of 30 is not 6 times better than one in an IF of 5. Yet they remain convinced that prepublication assessment of merit is the most appropriate means of assessment and that journal-level metrics, like the IF, provide the best surrogate. Because of the known biases with the IF, they suggest an alternative journal-level metric in the discussion, where journals are ranked by experts in different fields and ranks used as measure of an individual paper's merit.

This to us appears to contradict the central findings of the paper. It is not clear why experts should be more reliable at rating journals than rating articles. We would argue that prepublication reviewers are still influenced by the journal they are making the assessment for (e.g. potentially assessing different aspects of the work for “better” journals). Further, if our alternative interpretation of the findings is accepted, then any *binary* assessment (accept or reject) can only ever be a very weak indicator of the multivariate nature of a given paper's merit. Finally, as Bjoern Brembs and colleagues have argued in a recent review, given that the variance in article quality within any given journal is generally larger than any signal a quantitative journal quality measure can provide, any journal-based ranking (not just the IF) is potentially detrimental to science [10].

Indeed, any single metric that is highly variable is going to pose a problem for research assessment if we don't understand what is driving that variation. This is compounded when assessments are based on subjective opinion or other very biased measures, such as the IF. There is a sane solution, however, and that is to have a system of assessment that doesn't rely on one measure but uses a suite of metrics at the level of the article. In such a system it will also be important to enable research into new metrics of assessment. Crucial to this is the availability of data about research assessment itself. Although the Wellcome Trust and F1000 data used in this study are freely available (via Dryad [11]), the data upon which the RAE is based in the UK (to be known as the Research Excellence Framework, REF, in the next 2014 round) are not even collated, let alone available for others to analyse (assessors are asked to destroy their own raw assessment data). Eyre-Walker and Stoletzki recommend that all submissions to the UK REF be independently assessed by two assessors and then analysed. Likewise, similar data from grant panels or tenure decisions, wherever they are based, should be archived and made available for others to mine (while ensuring appropriate levels of confidentiality about individuals).

It is only with the development of rich multidimensional assessment tools that we will be able to recognise and value the different contributions made by individuals, regardless of their discipline. We have sequenced the human genome, cloned sheep, sent rovers to Mars, and identified the Higgs boson (at least tentatively); it is surely not beyond our reach to make assessment useful, to recognise that different factors are important to different people and depend on research context.

What can realistically be done to achieve this? It doesn't need to be left to governments and funding agencies. PLOS has been at the forefront of developing new Article-Level Metrics [12]–[14], and we encourage you to take a look at these measures not just on PLOS articles but on other publishers' sites where they are also being developed (e.g. *Frontiers* and *Nature*). Eyre-Walker and Stoletzki's study looks at only three metrics – postpublication subjective assessment, citations, and the IF. As one reviewer noted, they do not consider other article-level metrics, such as the number of views, researcher bookmarking, social media discussions, mentions in the popular press, or the actual outcomes of the work (e.g. for practice and policy). Start using these where you can (e.g. using ImpactStory [15],[16]) and even evaluate the metrics themselves (all PLOS metric data can be downloaded).

You can also sign the San Francisco Declaration on Research Assessment (DORA [17]), which calls on funders, institutions, publishers, and researchers to stop using journal-based metrics, such as the IF, as the criteria to reach hiring, tenure, and promotion decisions, but rather to consider a broad range of impact measures that focus on the scientific content of the individual paper. You will be in good company—there were 83 original signatory organisations, including publishers (e.g. PLOS), societies such as AAAS (who publish *Science*), and funders such as the Wellcome Trust.

Initiatives like DORA, papers like Eyre-Walker and Stoletzki's, and the emerging field of “altmetrics” [18]–[25] will eventually shift the culture and identify multivariate metrics that are more appropriate to 21^st^ Century science. Do what you can today; help disrupt and redesign the scientific norms around how we assess, search, and filter science.
